# Charge Transfer of Metal Porphyrins on a NaCl Thin Film Observed by Scanning Tunneling Microscopy in the Transport Gap

**DOI:** 10.1021/acsnano.5c01235

**Published:** 2025-05-07

**Authors:** Li-Qing Zheng, Abhishek Grewal, Kelvin Anggara, Fábio J. R. Costa, Christopher C. Leon, Klaus Kuhnke, Klaus Kern

**Affiliations:** † 28326 Max-Planck-Institut für Festkörperforschung, 70569 Stuttgart, Germany; ‡ Institut de Physique, École Polytechnique Fédéral Lausanne, 1015 Lausanne, Switzerland; § Gleb Wataghin Institute of Physics − University of Campinas−UNICAMP, Campinas 13083-859, Brazil

**Keywords:** scanning tunneling microscopy, charge transfer, transport gap, metal porphyrins, electronic decoupling

## Abstract

Elucidating the electronic structure of organic molecules in contact with a dielectric layer is essential to understanding and controlling many important processes, such as catalysis, photochemistry, and electroluminescence. However, this challenge calls for a detailed characterization of molecule-dielectric contacts on the atomic scale. Here, we employ scanning tunneling microscopy (STM) at low temperature (4 K) in combination with ab initio calculations to investigate the subnanometer-scale electronic states of photoactive molecules on a dielectric surface. For platinum and palladium octaethylporphyrin (PtOEP and PdOEP) adsorbed on few layers of NaCl on a metal substrate, our STM imaging of them in the energy gap between the frontier orbitals demonstrates their high sensitivity to the local environment, namely, adsorption site and applied voltage. Our calculations reveal that the states in this energy gap originate from combinations of molecular orbitals far from the Fermi level and that they are affected by the extent of molecule–surface partial charge transfer, which is tuned by adsorption site and voltage in the tunnel junction.

## Graphical abstract

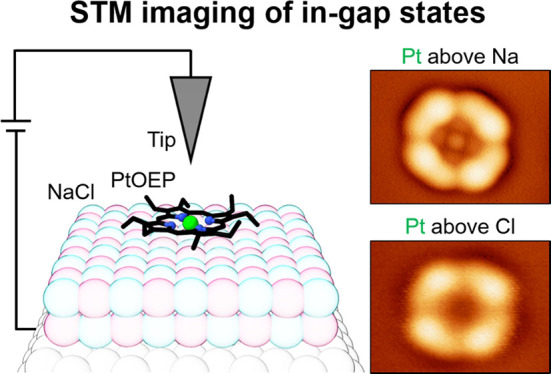

## Introduction

The optoelectronic properties of a molecule are often determined by the highest occupied molecular orbital (HOMO) and the lowest unoccupied molecular orbital (LUMO) of a molecule.^1–5^ Molecular classes exploited for these properties are, for example, metal porphyrins (MPs) and phthalocyanines (MPcs), widely used in biological, chemical, and device applications.^6–10^ Both MPs and MPcs can be driven by external electronic excitation as optoelectronic devices, as in organic light-emitting diodes (OLEDs). It is known that under these conditions, the frontier orbitals of the molecules play an important role. However, much less is known about the importance of the electronic gap between these orbitals, although it can be readily addressed by scanning tunneling microscopy (STM). This method has already been extensively employed to study the electronic density of states (DOS) and even the optical properties of single molecules with atomic spatial resolution.^11–15^ To avoid strong electronic hybridization of a molecule with a necessary underlying conductor (e.g., a metal), an atomically thin insulating layer between the molecule and substrate has been introduced, often realized by a few monolayers of NaCl.^14,16–19^ The first study using STM to explore electronically decoupled molecules with this approach was published in 2005.^20^ In that study, the HOMO, LUMO, and in-gap images of pentacene were recorded using STM. Although attracting at that time less attention than the frontier orbitals, the in-gap image can provide valuable information about the molecule. It can, for example, address the charge state of molecules^24,25^ which is of relevance in emerging atomic-scale nanotechnologies.^16,21–23^ Moreover, STM-induced charge injection into the HOMO–LUMO-gap of a molecule has been shown to be sufficient to excite molecular luminescence.^26^ A recent groundbreaking theoretical analysis revealed that charge transport within the gap between HOMO and LUMO involves not only the frontier orbitals but also molecular orbitals lying much lower in energy.^27^ This finding also explains why in-gap images may have simple geometries such as a featureless cross (for Pc) or a rectangle (for pentacene). Nevertheless, the effect of the local environment (defects, adsorption sites, and electric field) on the in-gap images of molecules remains to be investigated.

Here, we conduct a study that contributes to a better understanding of in-gap images of substituted MPs, namely, PtOEP and PdOEP photosensitizers.^28^ Due to their nonplanar character, they can realize different adsorption geometries on a dielectric layer and thus yield valuable information, for instance, how in-gap states are influenced by molecular geometries and by the interaction with an underlying NaCl lattice. Building on the theoretical insights from an earlier study,^27^ we find rich molecular features by examining in-gap images of PtOEP and PdOEP. First, the in-gap structure reveals the geometric size of a molecule more closely than its often distorted HOMO or LUMO images.^29^ Second, the chiral arrangement of the ligands becomes extremely pronounced. Third, the high intramolecular contrast of the electron transmission efficiency is revealed. Most importantly, we find a strong dependence of molecular features on the extent of molecule–surface charge transfer and demonstrate how to tune it in an STM.

## Results and Discussion

### PtOEP on 2 ML NaCl on Ag (111)

In order to prepare the samples, first, Ag (100), Ag (111), and Au (111) single crystals are partially covered by evaporating multilayers of NaCl. Next, a low (percent of a monolayer (ML)) coverage of PtOEP or PdOEP on a substrate precooled to ca. 100 K temperature is prepared by thermal evaporation in a UHV chamber (base pressure ∼ 10^–10^ mbar). For some samples, ZnPc is coevaporated to serve as a spectator molecular reference (Figure 1b,c). The sample is then transferred into the STM operated at liquid He temperature (4.2 K).^30^ For further details, see the experimental section in the Supporting Information.

**Figure 1:**
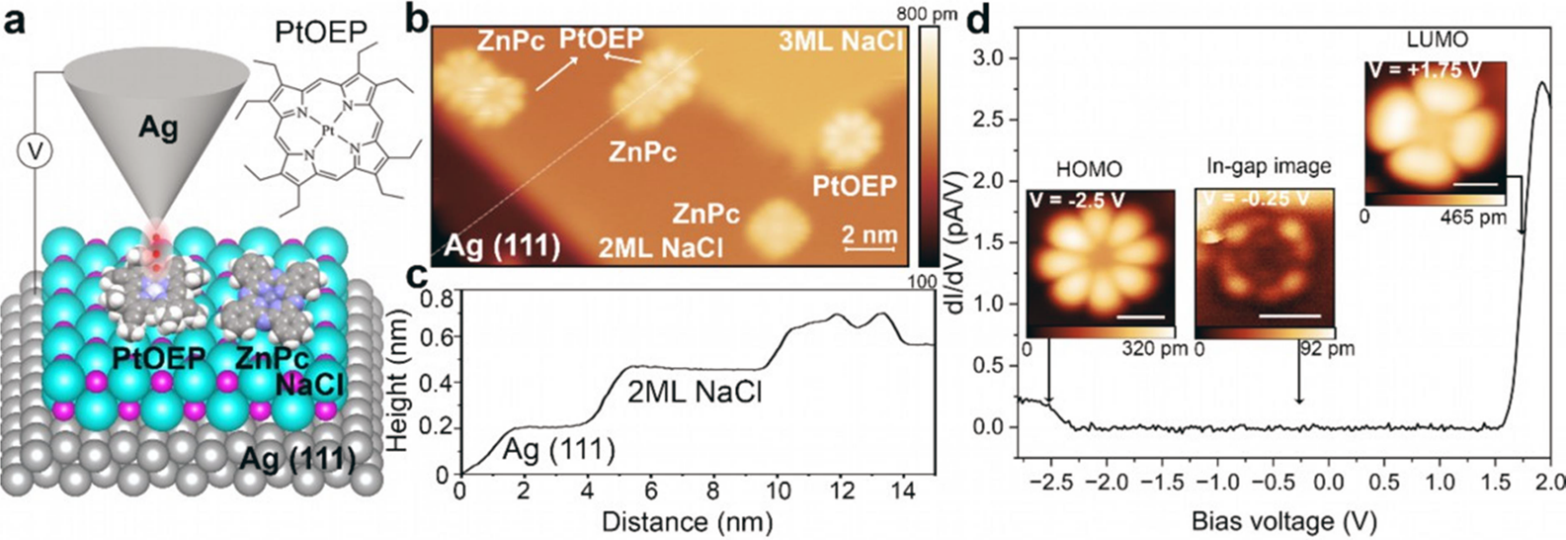
(a) Schematic of the experiment that probes the electronic states of PtOEP using STM imaging. Inset: Chemical structure of PtOEP. (b) STM image of PtOEP and ZnPc molecules coadsorbed on 2 ML NaCl/Ag (111); *V* = −2.5 V, *I* = 2 pA. (c) Height profile along the dashed white line in (b), intersecting a ZnPc attached to a PtOEP molecule that is attached to a NaCl step edge. PtOEP appears 0.05 nm higher than ZnPc and exhibits a stronger intramolecular contrast. (d) d*I*/d*V* spectrum (black curve) of a PtOEP molecule adsorbed on 2 ML NaCl/Ag (111). Insets: HOMO, in-gap, and LUMO images of a PtOEP molecule acquired at the indicated voltages. Scale bars: 1 nm. The color scale for the height information is given below each image.

Inspection of the 2 ML NaCl sample in the STM finds PtOEP/ZnPc dimers and individual PtOEP molecules adsorbed at salt edges (Figure 1b), indicating still a high mobility of PtOEP on NaCl at the deposition temperature of 100 K. The differential conductance (d*I*/d*V*) spectrum of a PtOEP molecule is shown in Figure 1d. The spectrum exhibits two sharp edges at −2.28 and +1.56 V, which correspond to the onset of resonant tunneling into the positive and negative ion resonances of PtOEP. STM images acquired on these resonances exhibit the HOMO and LUMO (insets in Figure 1d) in good agreement with the simulated orbital images (Figure S1a,b). The middle inset in Figure 1d is acquired within the transport gap at −0.25 V, showing for the first time the in-gap STM image of PtOEP. The image features inner and outer ring sections. In the outer ring, each section is observed to increase in height in the anticlockwise direction, thus revealing the chiral geometry of the molecule. This observed chirality is ascribed to the ethyl groups alternating in height (by ∼18 pm). In the inner ring, four circular features are observed. Our experimental results show that the HOMO and LUMO images of PtOEP are qualitatively different from its in-gap images, as confirmed by the DFT calculations (Figures S1 and S2). The apparent height of the features in the STM images of in-gap states lies several Angstroms lower than that of the HOMO and LUMO images, corresponding to 2–4 orders of magnitude lower in current when considered under constant height conditions. We accordingly find that the STM images of PtOEP acquired at −2.5 V (Figure S2a) and +1.6 V (Figure S2i) are not perceptibly modified by the in-gap states and agree well with the calculated HOMO and LUMO images of PtOEP.

A closer examination of the PtOEP in-gap images reveals dark rims, outlining the interior and exterior of the ring-like structure that become more prominent with decreasing bias voltage (Figure S2b–h). Dark regions indicate areas where the STM tip must approach the molecule in order to keep the tunnel current constant. This means that electron transport through the border of the molecule is less efficient than that through the pristine NaCl film. Such suppression of electron transmission is also evident in certain regions of the molecule (see, for example, the experimental image in Figure 4b). Both indicate a reduced electronic DOS. As these features are, however, much less pronounced in the DFT calculations (which are sensitive to DOS effects, Figure 4), these features may be enhanced by interference effects between different electron transmission pathways.^31^

### PtOEP on 2 ML NaCl on Au (111)

Next, we use Au (111) as a substrate with a higher work function than Ag (111), and we observe subtle changes of the in-gap images of PtOEP. Figure 2 shows STM images and a differential conductance spectrum of a PtOEP molecule on 2 ML NaCl on Au (111). Due to the increased substrate work function, the energies of HOMO and LUMO edges are shifted to higher energy by 1.16 and 0.8 eV, respectively, when compared to Figure 1d. The minor change in the electronic bandgap (0.28 eV) is ascribed to the different local environment of the molecule.^17^ Next, in-gap patterns of the molecule are recorded for bias voltages between −1.25 and +1.5 V (Figure 2 bottom panel). The HOMO image of the molecule (at −1.25 V) has not changed in comparison with the inset of Figure 1d. Moreover, the in-gap structure of the molecule still exhibits four outer and inner ring sections, as observed on Ag (111). However, in all in-gap images, the center of the molecule features a peculiar protrusion that varies in height depending on the applied bias voltage. This central protrusion is not primarily due to the changed metal substrate, because we also find PtOEP molecules on 2 ML NaCl on Au (111) with similar features to the one shown in the inset of Figure 1d (see Figure S3). Constant-height STM images of the HOMO and LUMO of PtOEP on 2 ML NaCl on Au (111) are also provided (see Figure S4), where the effect of the feedback loop on the imaging can be excluded. Due to the exponential dependence of current on distance, conductance map images exhibit currents varying over orders of magnitude and thus lose some details of the topographic features. While the LUMO images in constant height and constant current mode are qualitatively similar, we find that due to the increased contrast, the height alternation in the lobes of PtOEP appears more clearly in the constant-height image (Figure S4a). We applied a basic asymptotic approximation to the protrusion height of the in-gap structures, and we observed a deviation from linearity around 0 V. Notably, the protrusion height exhibits a nonlinear voltage dependence, suggesting the interplay of multiple physical parameters, such as electric field distribution, tip geometry, and tunneling current. Although these observations highlight the complexity of the underlying physics, a comprehensive theoretical interpretation would require advanced modeling, which is beyond the scope of this study.

**Figure 2:**
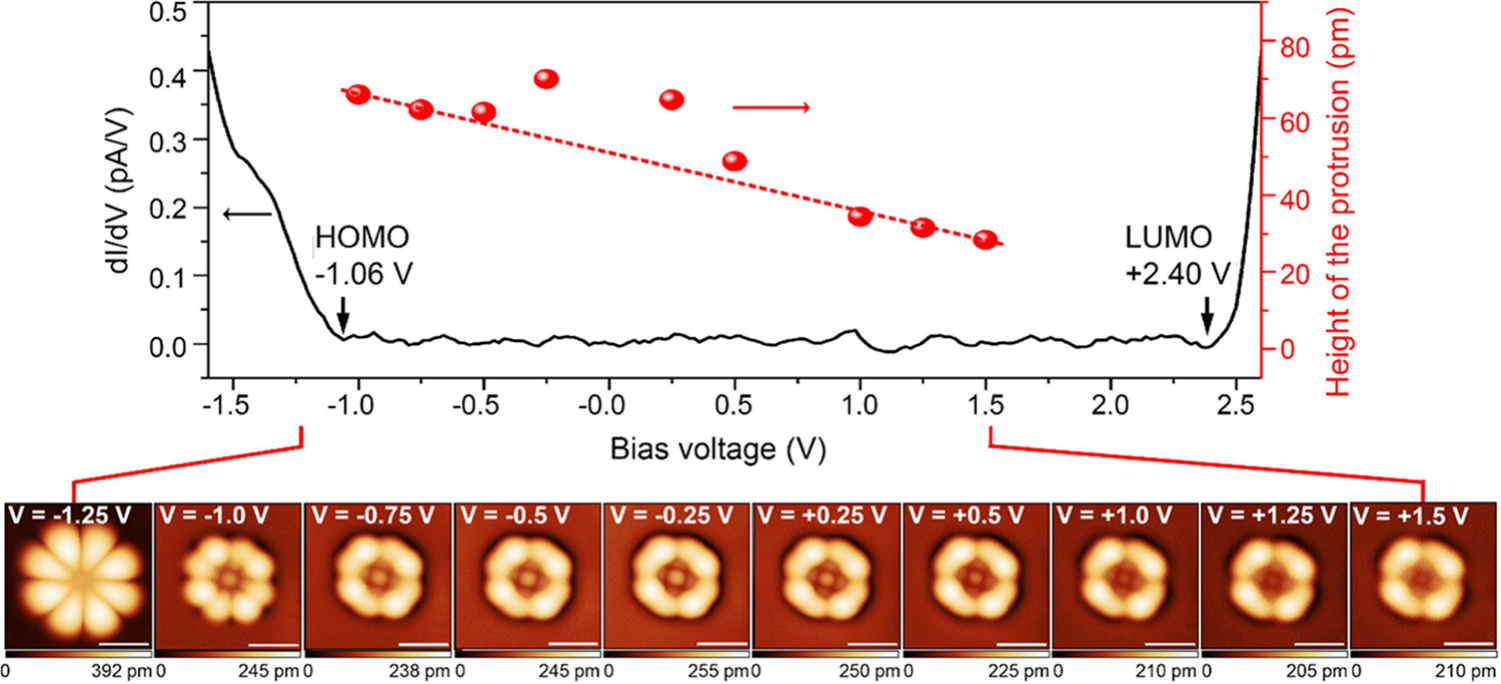
Top panel: d*I*/d*V* spectrum (black curve) of a PtOEP molecule on 2 ML NaCl/Au (111) and plot of the height (red symbols) of the central protrusion of the molecule obtained by the evaluation of the images below. The asymptotic behavior of the protrusion for higher positive and lower negative voltages is indicated by the red dashed line. Bottom panel: STM images were acquired at the indicated voltages. Scale bars: 1 nm. The color scale coding for the height is given below each image. For details of the evaluation, see the text in the Supporting Information Section 1.3 and Figures S11–S12.

### Modification of the In-Gap Image by Adsorption Site and Center Metal Atom

To understand the origin of the center structure in the in-gap image of PtOEP, we first focus on the appearance of the molecular center on different adsorption sites on the NaCl lattice. High-resolution STM images of an area with PtOEP and ZnPc molecules on 2 and 3 ML NaCl on Ag (111) are shown in Figure 3. The topmost PtOEP molecule adsorbed at the right NaCl edge (PtOEP **1**) exhibits a central protrusion, while the other two (PtOEP **2** and PtOEP **3**) have a dark center (Figure 3a). As demonstrated previously, the protrusions of the NaCl lattice always represent the Cl lattice site.^32^ Thereby, based on the STM image (Figure 3b), we determine that PtOEP **1** is adsorbed with its metal core located on top of a Na ion, while PtOEP **2** and PtOEP **3** are adsorbed with their center on top of Cl ions. We thus identify that a protrusion observed in the middle of the in-gap structure indicates that the center of the PtOEP is located on top of a Na ion. Further evidence is shown in Figure S5 of the Supporting Information. Based on the DFT calculations introduced below, we can ascribe the different center structure of the in-gap imaging to the interaction between the metal ions and NaCl.

**Figure 3:**
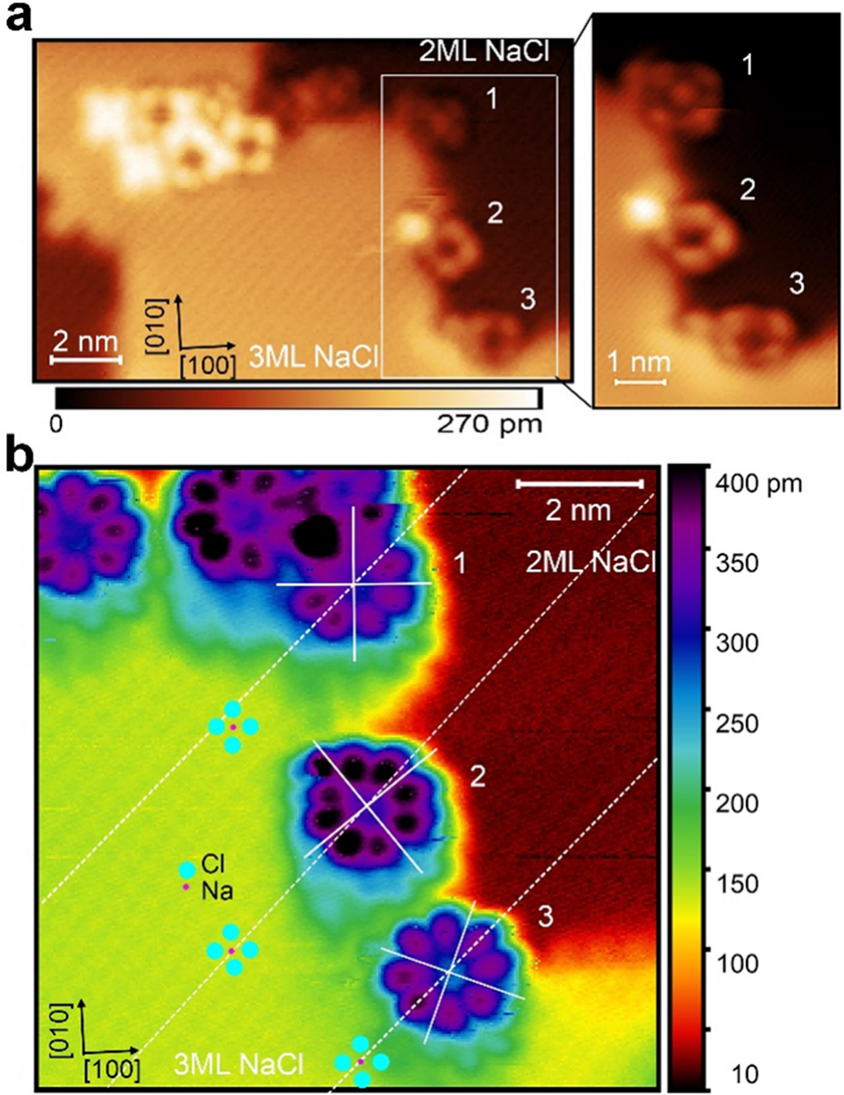
(a) In-gap STM image showing PtOEP and ZnPc molecules coadsorbed on NaCl/Ag (111). Inset: zoom-in on the three PtOEP molecules showing different in-gap structures (*V* = −1.5 V, *I* = 2 pA). (b) STM image acquired in the same area as panel (a) (*V* = −2.5 V, *I* = 2 pA). The red and blue dots in (b) mark the positions of Na and Cl ions of the NaCl (100) lattice, respectively. The white lines indicate the center and orientation of the molecules, and the white dashed lines follow the atomic rows of the NaCl lattice. Note that molecules 1, 2, and 3 are adsorbed on the lower terrace at a NaCl step.

Next, we examine the role of the center metal atom in the in-gap imaging. We used a sample prepared with a molecule featuring a different metal center: PdOEP. Compared to the Pt ion, the Pd ion has a different *d* electron configuration (and d orbitals), which we anticipate to interact differently with NaCl. An equivalent evaluation of the PdOEP adsorption site and central protrusion finds that the appearance is exactly opposite to the one of PtOEP, that is, adsorption on Na yields a dark center, while adsorption on Cl yields a bright center. For the detailed evaluation, see Figure S6 in the Supporting Information. Note that PdOEP is adsorbed on 2 ML NaCl on Ag (100). The HOMO image of PdOEP on 2 ML NaCl on Ag (111) and the bias spectrum of PdOEP are shown in Figure S7. Compared to Figure S6a, the HOMO image of PdOEP on 2 ML NaCl on Ag (111) is nearly identical with that on 2 ML NaCl on Ag (100) (Figures S6 and S7). Moreover, the HOMO and in-gap images of PdOEP are comparable to those of PtOEP on 2 ML NaCl on Ag(111) and Au(111), and no perceptible difference in the feature of their HOMO and in-gap images is observed for the different metal substrates. Thus, in line with the previous discussion of PtOEP, we conclude that the HOMO, LUMO, and in-gap images of PtOEP and PdOEP are unaffected by the choice of metal substrates underneath the thin NaCl layers.

### DFT Calculations

To understand the various in-gap structures obtained in our experiments, we performed DFT calculations of the HOMO, LUMO, and in-gap images of PdOEP and PtOEP on 2 ML NaCl on Ag substrates. The simulated images (Figure 4a,b, SIMUL) reproduce the observed dependence of in-gap images on adsorption sites and metal centers. Consistent with the experiment, Figure 4a shows that the Pt in PtOEP appears bright when situated above a Na ion and dark when situated above a Cl ion, whereas Figure 4b shows that the Pd in PdOEP appears dark above Na and bright above Cl. Notably, these properties are found to be unaffected by a rotation of the molecule (simulations not shown). From our analysis, we determine that these in-gap appearances originate from the hybridized states formed between the underlying surface states and the molecular orbitals (Figure S9), particularly unfilled orbitals far beyond the LUMO (Figure S10), similar to Xe on Ni (110).^33^ In addition, our calculations show that these hybridized states can form nodal-plane-like features on the surface around the molecule that appear in our simulation as a dark rim on the surface around the protrusion (SIMUL panels in Figure 4), in agreement with the experimental observations (EXPT panels in Figure 4).

**Figure 4:**
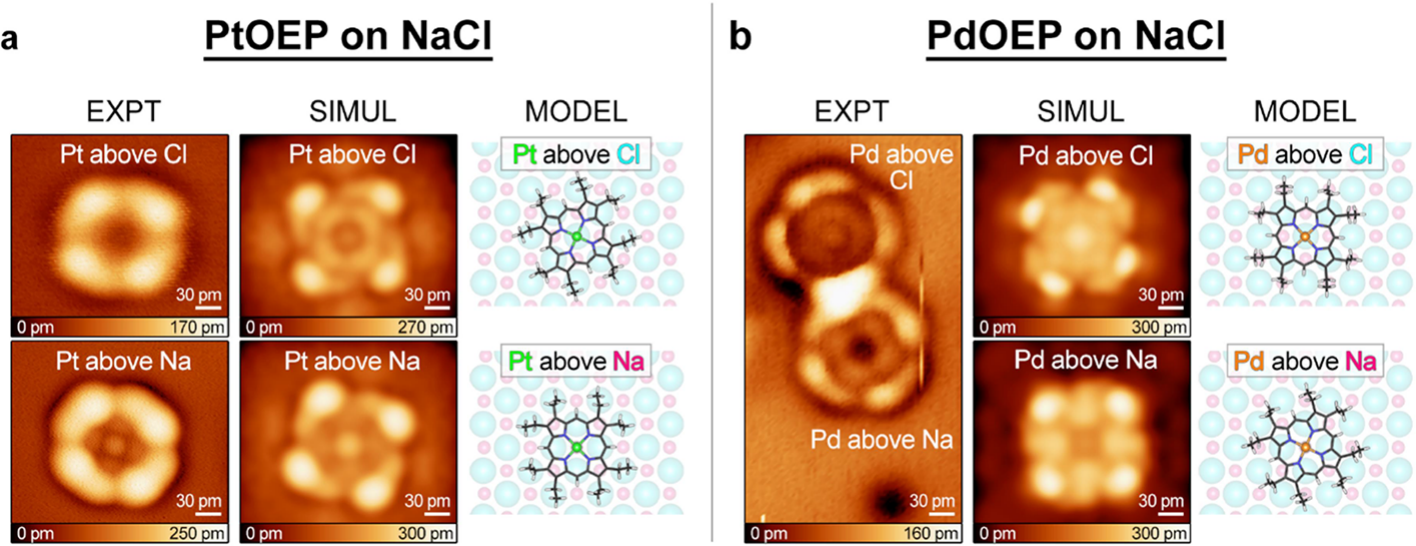
Experimental in-gap images of PtOEP (a) and PdOEP (b) on NaCl adlayer at metal surfaces are reproduced by simulated STM images from the DFT calculations of PtOEP on 2 ML NaCl on Ag(111) and PdOEP on 2 ML NaCl on Ag(100) (see Methods for calculation details and Figure S8).

Our calculations reveal further insights into the in-gap appearance of metal atoms in PdOEP and PtOEP when absorbed above a Na ion or a Cl ion. Our charge analysis (Figure 5b) shows that, when Pd is located above a Cl ion, the Pd 4d_*z*_^2^ receives an electron from the surface; whereas, when Pd is located above an Na ion, the Pd 4d_*z*_^2^ donates an electron to the surface. Notably, this Pd 4d_*z*_^2^ orbital is the only Pd state within the PdOEP HOMO–LUMO gap that strongly contributes to the in-gap appearances of the Pd atom in PdOEP (Figure S9). Our analysis of the projected density-of-states (pDOS) shows that when Pd is above a Na ion, Pd­(4d_*z*_^2^)-to-surface electron transfer lowers the 4d_*z*_^2^ energy (attributable to the lower electron–electron repulsion in the 4d orbitals) and thereby reduces the 4d_*z*_^2^ contribution to the in-gap states, resulting in the dark STM in-gap appearance of Pd. Conversely, when Pd is above a Cl ion, small surface-to-Pd­(4d_*z*_^2^) electron transfer maintains the 4d_*z*_^2^ energy close to the Fermi level and thereby maintains the significant 4d_*z*_^2^ contribution to the in-gap states, resulting in the bright STM in-gap appearance of Pd. Our analysis underscores the significant role of molecule–surface electron transfer in the in-gap states of molecules on dielectric layers.

**Figure 5:**
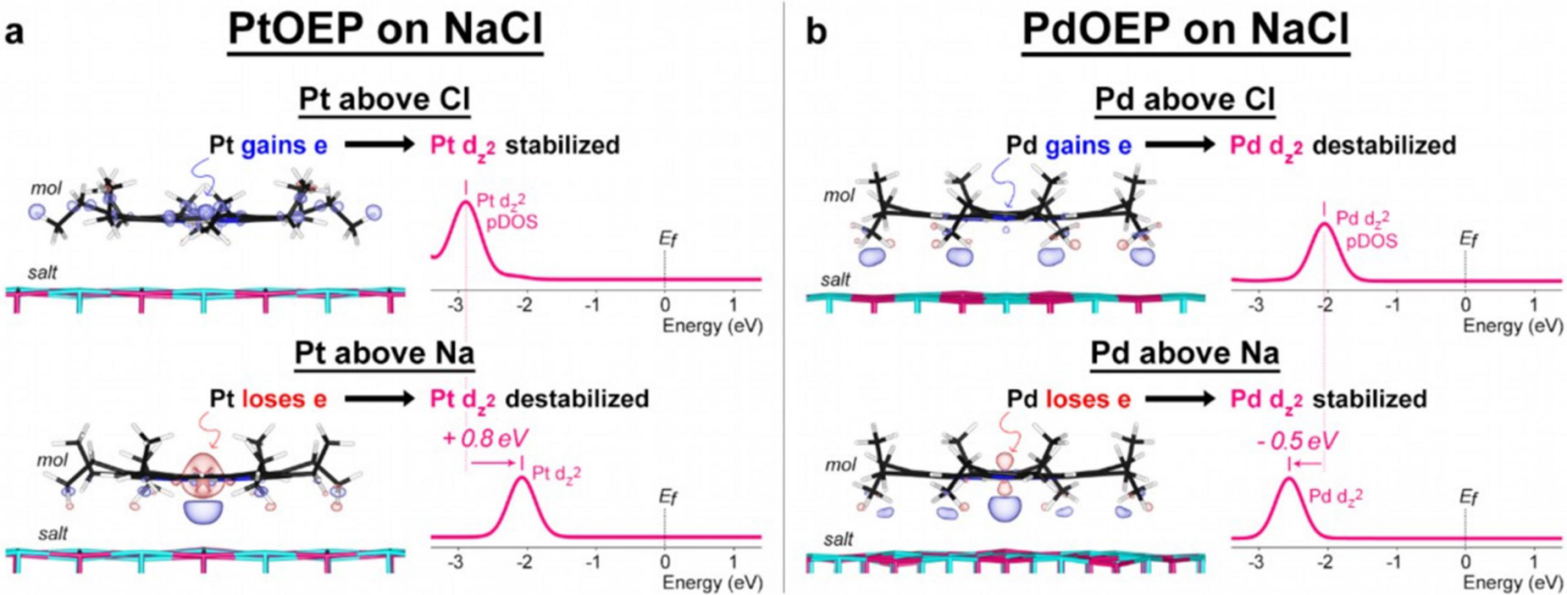
Adsorption site of the metal atom in PtOEP (a) and PdOEP (b) varies the molecule–surface partial electron transfer that changes the metal d-orbital energy levels and thus the in-gap appearance of the metal atom. For each case, the charge transfer density shows whether the metal atom in the molecule gains partial charge (in blue) from the surface or loses partial charge (in red) to the surface. The isosurface shown for PtOEP charge transfer density is 2 × 10^–3^ eÅ^–3^ and, for PdOEP, 1.5 × 10^–3^ eÅ^–3^. On the right-hand side of each panel, the pDOS shows the effect of such molecule–surface partial charge transfer in shifting the energies of Pt 5d_*z*_^2^ and Pd 4d_*z*_^2^ orbitals that are responsible for the in-gap appearance of Pt in PtOEP and Pd in PdOEP.

Similar analysis performed on PtOEP (Figure 5a) reveals molecule–surface electron transfers similar to those of PdOEP (Figure 5b), but with a more complex response in Pt states. Our charge analysis shows the principal Pt state interacting with the surface to be a complex mixture of Pt 5d orbitals (Figure 5a), which may be due to the increased electron correlation effects or the relativistic effects in 5d metals.^34,35^ As a result, the energy shifts of the Pt 5d_*z*_^2^ orbital, which strongly contribute to the in-gap appearance of Pt in PtOEP, differ significantly from the energy shifts in Pd 4d_*z*_^2^ in PdOEP. When Pt is above a Cl ion, surface-to-Pt­(5d_*z*_^2^) electron transfer lowers the 5d_*z*_^2^ energy and thus reduces the 5d_*z*_^2^ contribution to the in-gap states, resulting in the dark STM in-gap appearance of Pt. Conversely, when Pt is above a Na ion, Pt­(5d_*z*_^2^)-to-surface electron transfer puts the 5d_*z*_^2^ close to the Fermi level, resulting in the bright STM in-gap appearance of Pt. Our DFT calculations thereby reveal at the atomic level how in-gap states are influenced by molecule–surface interactions.

### Reversible Manipulation and Monitoring of the PtOEP Adsorption Site

Figure 6 shows an arrangement in which a PtOEP molecule is stabilized by two nearby ZnPc molecules on 3 ML NaCl on Ag (111). Starting with the molecular positions shown in the left panel, d*I*/d*V* spectroscopy is performed, leading to the displacement (and rotation) of the PtOEP and one of the ZnPc molecules (middle panel). After increasing the STM bias voltage to +2.5 V for a few seconds, the original positions are restored (right panel). The displacement vector of the PtOEP molecule, 0.28 nm approximately in the [010] direction of the NaCl lattice, indicates a switching from a Cl adsorption site to a Na site and back, confirmed by the observation that the center of PtOEP changes brightness, from dark to bright and after the second step back to dark again. This demonstrates the reversible manipulation of PtOEP between adsorption sites and its supervision by means of the central feature’s appearance. The full experimental data series and the bias spectra of these molecules are displayed in Figures S13–S15 in the Supporting Information.

**Figure 6:**
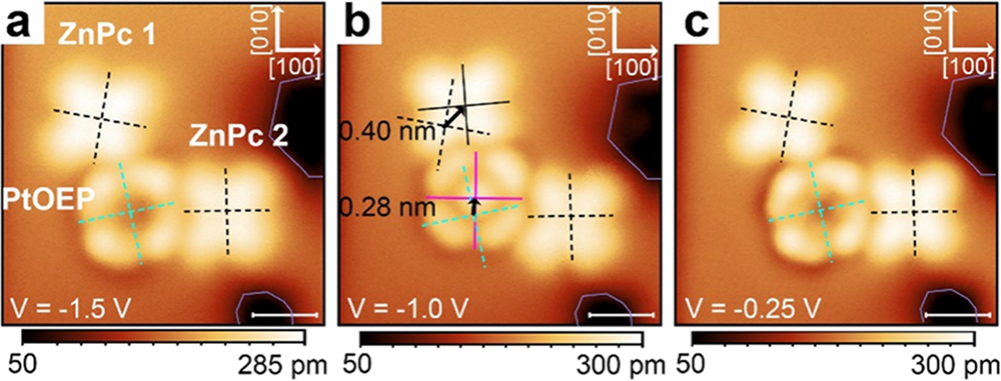
(a–c) STM images of a PtOEP and two ZnPc molecules coadsorbed on 3 ML NaCl/Ag (111) (*I* = 2 pA) acquired before (a) and after performing bias spectrum measurements over the range from *V* = −2.5 to +2.5 V (b) and after applying a voltage of +2.5 V for a few seconds (c). The voltage at which each image is recorded is given in each panel. The dashed and solid crosses mark the orientation and center of ZnPc (black) and PtOEP (green, red) molecules. Scale bars: 1 nm. The color scale for the height information is given below each image. This figure shows 3 panels from an extended data series shown in Figure S13 in the Supporting Information.

## Conclusions

STM in-gap images of PtOEP and PdOEP on thin NaCl films in combination with DFT calculations provide a powerful tool to access molecular properties such as structure, chirality, and local molecular charge. The calculations reproduce the in-gap STM images and reveal details of the observed charge transfer.

We investigate the link between the in-gap states of PtOEP and PdOEP and their external environment, represented by their adsorption sites on NaCl. We demonstrate that the interaction between metal ions and the NaCl film underneath is crucial for the molecule’s in-gap appearance, ascribed to the different responses of the Pd (4d_*z*_^2^) and Pt (5d_*z*_^2^) to the charge transfer with either a Na or a Cl ion. As a result, in PtOEP, Pt appears dark when it is located above a Na ion and bright when located above a Cl ion, while in PdOEP, it is the opposite. Notably, the apparent height of the metal in-gap appearance can be tuned by the voltage applied to the tunnel junction, which reduces or pronounces the charge transfer between the central metal atom in the molecule and the underlying surface. Molecular manipulation demonstrates a reversible switching of the PtOEP adsorption site, from the Na ion to the Cl ion and back, resulting in characteristic in-gap structures that confirm their strong dependence on the adsorption site. This study illustrates the capability of STM imaging within the electronic transport gap to access the essential physical properties of molecules adsorbed on a dielectric.

## Supplementary Material



## Notes

Details of sample preparation and density functional calculations, evaluation of the central protrusion height with bias voltage, simulated STM images of the frontier orbitals of PtOEP and ZnPc on 2ML NaCl on Ag (111); constant current STM images acquired at negative and positive bias voltages; constant-height STM images of PtOEP on 2ML NaCl on Au (111); high-resolution STM image of PtOEP and ZnPc on 2 MLs of NaCl on Ag (111); STM image of the HOMO of two PdOEP molecules adsorbed on 2 ML NaCl/Ag (100); d*I*/d*V* spectra of PdOEP on 2ML NaCl/ Ag (111); simulated STM images of the frontier orbitals and in-gap state of PdOEP adsorbed on a Cl and a Na ion; overlap analysis of PdOEP; evaluation of the central protrusion; alternative evaluation of the data; STM images acquired at negative and positive bias voltages of PtOEP and ZnPc molecules; and d*I*/d*V* spectra of the PtOEP molecule (PDF)

## ∥

State Key Laboratory of Analytical Chemistry for Life Science, School of Chemistry and Chemical Engineering, Nanjing University, Nanjing 210023, People’s Republic of China

## ⊥

Département de chimie, Université Laval, Québec, Québec G1 V 0A6, Canada.

## Abbreviations

PtOEP: platinum octaethylporphyrin
PdOEP: palladium octaethylporphyrin
ML: monolayer
ZnPc: zinc phthalocyanine
STM: scanning tunneling microscopy
DFT: density functional theory
