# Supplementary material for: Charge Transfer of Metal Porphyrins on a NaCl Thin Film Observed by Scanning Tunneling Microscopy in the Transport Gap
Source: ACS Nano. 2025 May 7;19(19):18357–63. doi: 10.1021/acsnano.5c01235 (PMC12096429; doi:10.1021/acsnano.5c01235)
Supplement: Supplementary file 1 [file nn5c01235_si_001.pdf]

Supporting information

Charge transfer of metal porphyrins on a NaCl thin film observed by scanning tunneling microscopy in the transport gap

Li-Qing Zheng[1*†], Abhishek Grewal[1], Kelvin Anggara[1*], Fábio J. R. Costa [1,3], Christopher C. Leon[1‡], Klaus Kuhnke[1*], Klaus Kern[1,2]

1. Max-Planck-Institut für Festkörperforschung, Heisenbergstraße 1, 70569 Stuttgart, Germany
2. Institut de Physique, École Polytechnique Fédéral Lausanne, 1015 Lausanne, Switzerland
3. Gleb Wataghin Institute of Physics - University of Campinas – UNICAMP, Campinas 13083-859, Brazil

Present addresses:
†: State Key Laboratory of Analytical Chemistry for Life Science, School of Chemistry and Chemical Engineering, Nanjing University, Nanjing 210023, People's Republic of China.
‡: Département de chimie, Université Laval, Québec, Québec G1V 0A6, Canada

1. Supplementary text

1.1 Sample preparation and STM measurements

All experiments are performed with a home-built low temperature ultrahigh-vacuum STM operated at 4.2 K (<10^{-11} mbar).[1] Prior to use, Ag (100), Ag (111), and Au (111) single crystals are cleaned by argon ion sputtering and subsequent annealing to 660K, 670 K and 873 K, respectively, for several cycles. NaCl is evaporated thermally from a Knudsen cell held at 900 K, with the Ag (100), Ag (111) or Au (111) surface held at 300 K, to obtain a partial coverage of defect-free and (100)-terminated NaCl islands. After NaCl evaporation, the substrate is annealed at 320K for 10 min, in order to obtain large islands with 2-4 layers thickness. Finally, PdOEP or PtOEP and ZnPc molecules are thermally evaporated at a sample temperature of ca. 100 K. The overall molecular coverage is typically in the lower percent range. Electrochemically etched Ag tips[2] are used in all experiments. They are cleaned by argon ion sputtering to remove oxides, while electrochemically etched Au tips[3] are used without argon sputtering treatment. To clean a tip, further tip modification through voltage pulses or tip indentation is regularly used. STM imaging and spectral measurements are taken at 4.2 K in constant-current or constant-height mode. Text and figure cations specify bias voltages of the metal substrate with respect to the grounded tip and indicate the employed tunneling current. Differential conductance (dI/dV) spectra are measured using standard digital lock-in techniques with a bias modulation of V_{rms} = 10 mV at 629 Hz provided by the Nanonis software that is employed for all measurements.

Notably, in contrast to metal phthalocyanines (MPcs), PtOEP and PdOEP molecules are less stable on the NaCl layers, due to the steric hinderance of the ethyl groups. As will be discussed below, the ligands in PtOEP and PdOEP are found to point up and down alternatingly when adsorbed on the surface, which weakens the contact between the molecules and NaCl layer and reduces the attractive van der Waals forces. Although the molecules were evaporated on substrates kept at a relatively low temperature (ca. 100 K), they were found to be mobile when adsorbing on the NaCl surface. As a result, PtOEP and PdOEP molecules tend to agglomerate on the surface or attach to NaCl step edges (see Figure 1b). To reduce the instability of the molecules on the NaCl, the sample is studied at 4.2 K, and all STM images were acquired at low tunneling current (~2 pA).

1.2 Density functional theory (DFT) calculations

DFT calculations were performed to model the in-gap appearances of PdOEP and PtOEP. All structures were visualized using the VESTA software[4]. To model observations on surface, we used the Vienna Ab-initio Simulation Package (VASP, ver. 5.4.4)[5,6], implementing the projection-augmented wave function (PAW) method[7,8] with an energy cut-off of 400 eV, the Perdew-Burke-Ernzerhof (PBE) functional[9], and the Grimme's DFT-D3 VdW correction[10]. Using a supercell with 20 Å vacuum space and by sampling only the gamma points of the k-mesh, the relaxation calculations were performed until the forces were below 0.01 eV/Å for all atoms. The Ag-surfaces were modeled as a slab with 5 layers of Ag, while the NaCl layers were modeled as a bilayer.

For electronic calculations, we employed the Local Density Approximation (LDA) approach as the outcomes agreed better with experiments. STM simulations were obtained via Tersoff-Hamann method[11]. Projected density-of-state calculations employed Gaussian smearing with σ = 0.25 eV. In the case of PdOEP, we used the standard LDA approach without U corrections; whereas, in the case of PtOEP, we used the LDA+U approach, where we varied the U parameters to obtain STM simulations that reasonably reproduced the experimental STM images. We found that U = +15 eV was required for the case of Pt above Cl and U = -15 eV was required for the case of Pt above Na. Charge transfers between molecule and surface were obtained by evaluating the difference between total charge density of molecule on surface ($\rho_{mol+surf}$) and the sum between total charge density of molecule in gas (ρ_{mol}) and total charge density of surface without molecule (ρ_{surf}). Mathematically, the charge transfer density is $\rho_{mol+surf} - (\rho_{mol} + \rho_{surf})$.

Analysis of electronic structures were performed by evaluating overlap integral between two arbitrary states, mathematically expressed as:

$$\langle State\ 1 | State\ 2 \rangle = \int State\ 1^{*}\ \ State\ 2 \quad d\tau$$

Individual wavefunctions were extracted from the WAVECAR file (obtained from VASP calculations) using the pyvaspwfc package (https://github.com/liming-liu/pyvaspwfc). This analysis was performed to (1) determine the contribution of molecular orbitals (e.g. HOMO of PdOEP) to the in-gap states of the molecule on surface (e.g. PdOEP); and (2) determine the contribution of specific atomic orbitals (e.g. Pd $4d_{z^2}$) to the in-gap states of the molecule on surface (e.g. PdOEP).

1.3 Change of the central protrusion with bias voltage

The lower panel of Fig.2 of the main text shows constant current measurements of the in-gap topography as a function of voltage. The upper panel of Fig.2 plots the evaluated height difference between central protrusion and NaCl plane in the STM as a function of bias voltage. In the following we describe how the topography was evaluated. Fig. S11a shows the constant current topography measured at -1V as an example for the evaluation procedure. In order to reduce the scatter of the average height and the maximum height of the protrusion they are determined using automated averaging and fitting. The average NaCl z coordinate is obtained as the average of forward and backward scan outside the dashed square in Fig. S11a, corresponding to an average over 22500 pixels of the topographic data. This average yields the blue squares in Fig. S11c (bottom panel). The height of the protrusion on the metal center of the molecule is the maximum found by a 2-dimensional Gaussian fit, which extends over the central 15x15 pixels of the protrusion. In order to account for horizontal drift in the STM data, a first coarse fit determines the position of the maximum on which the second fit is then centered. Fig. S11b shows the horizontal central section through the molecule’s topography (red) and the section through the second 2D-Gaussian fit (blue). The red circles in Fig. S11c (bottom panel) represent the z coordinate of the central protrusion. The difference between blue and red z coordinates provide the relative height of the central protrusion above the NaCl surface plane (Fig. S11c top panel).

The black circles in Fig. S11c indicate (1) a negative slope with applied voltage, however, with a significant deviation from a straight line near zero voltage. This indicates (2) that the protrusion appears higher whenever the tip approaches to the molecule which happens at low absolute voltages as shown by the z coordinate of the protrusion. In Fig. S12, we analyzed the data by an alternative evaluation, measuring the protrusion height with respect to the dark molecular center instead of referencing the protrusion to the NaCl surface plane as we do in the manuscript. This evaluation confirms the height increase around zero bias, thus excluding that this effect is an artefact of the evaluation. An attempt to account for this behaviour as a pure electric field effect did, however, not provide a fully consistent picture. In Fig.2 we provide a straight line to indicate the asypmtotic behaviour as a function of applied voltage.

1.4 STM manipulation of a PtOEP molecule

We observed that PtOEP can become unstable at positive bias (see Fig. S15) and wondered if we can manipulate PtOEP to switch between the two inequivalent adsorption sites on Na and Cl by simply applying a positive bias voltage. In a data series of a PtOEP molecule on 3ML NaCl on

Ag (111), where PtOEP is more mobile than on 2ML NaCl, we stabilized it by two nearby ZnPc molecules. Fig. S10 shows a series of measurements at various sample voltages showing HOMO, in-gap, and LUMO features of PtOEP and ZnPc molecules. At -2.5 V for all 3 molecules, tunneling still takes place from the HOMO (Fig.S13a). At a bias of -2 V, ZnPc 2 shows its in-gap image (cross) while the HOMO is still seen for ZnPc 1 (Fig.S13b). This difference is ascribed to the nearby NaCl step edge which causes a small shift of the HOMO and LUMO levels of ZnPc 2 (see the dI/dV spectra in Figure S14). In Fig. S13 b,c, PtOEP exhibits its in-gap image with a dark center, indicating that the metal center is located on top of a Cl ion. To move the molecule, we perform a scan over the bias range -2.5 V to 2.5 V on one lobe of the PtOEP molecule. Subsequently, we record STM images at -1 V and +0.5 V, shown in Fig. S13 d,g. In contrast to Fig. S13 b,c, these STM images show the in-gap structure of PtOEP with a central protrusion apart from a minor reorientation. The center of PtOEP is shifted by 0.28 nm within experimental accuracy in the <010> direction of the NaCl lattice (Fig.5b) which allows the conclusion that the molecule shifted from a Cl to a Na site.[12-13] In contrast, the ZnPc moves by 0.40nm approximately along the [110] direction which can be ascribed to a motion between equivalent (Na or Cl) sites on the NaCl lattice.

Induced by the rotation of PtOEP, also ZnPc 1 rotates slightly to the left, yet without a change of its in-gap image. Since we know that PtOEP is unstable at voltages above +2 V, we deliberately apply to the molecule a bias of +2.5 V for a few seconds until a sudden change of current indicates another switch of adsorption site. After that event, STM images were recorded at -0.25 V, +0.25 V, +0.75 V (Fig. S13 e,f,h). By the second manipulation, the in-gap image of PtOEP changed back to the original one, documented in Fig. S13c. Actually, both, PtOEP and ZnPc moved back to their original position. This procedure demonstrates the successful manipulation of the PtOEP adsorption site from an Na ion to a Cl ion and back. Notably, at V = +0.75 V, the two ZnPc molecules already exhibit their LUMO, so that the low profile of the in-gap structure of PtOEP is partially obscured by the bright orbitals of the neighboring ZnPc molecules. Finally, at V > 1.5 V tunneling occurs into the LUMO for all three molecules (Fig. S13 I,j). Note, that at V > +1.5 V the molecule has rotated by 45° due to its instability at positive bias.

2 Supplementary figures

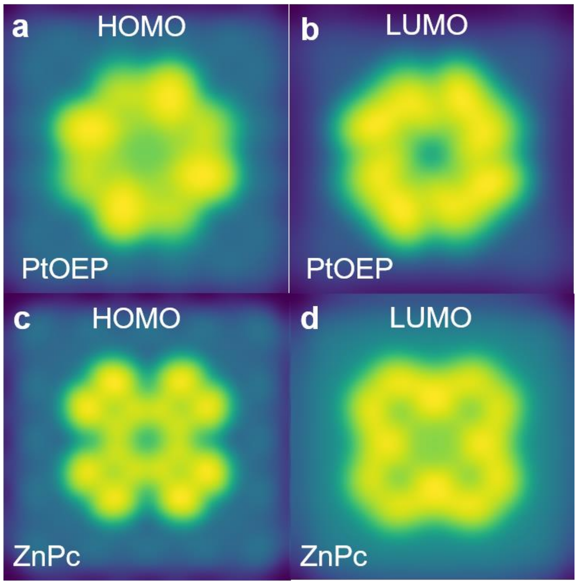

Figure S1. Simulated STM images of the frontier orbitals of PtOEP and ZnPc on 2ML NaCl on Ag (111).

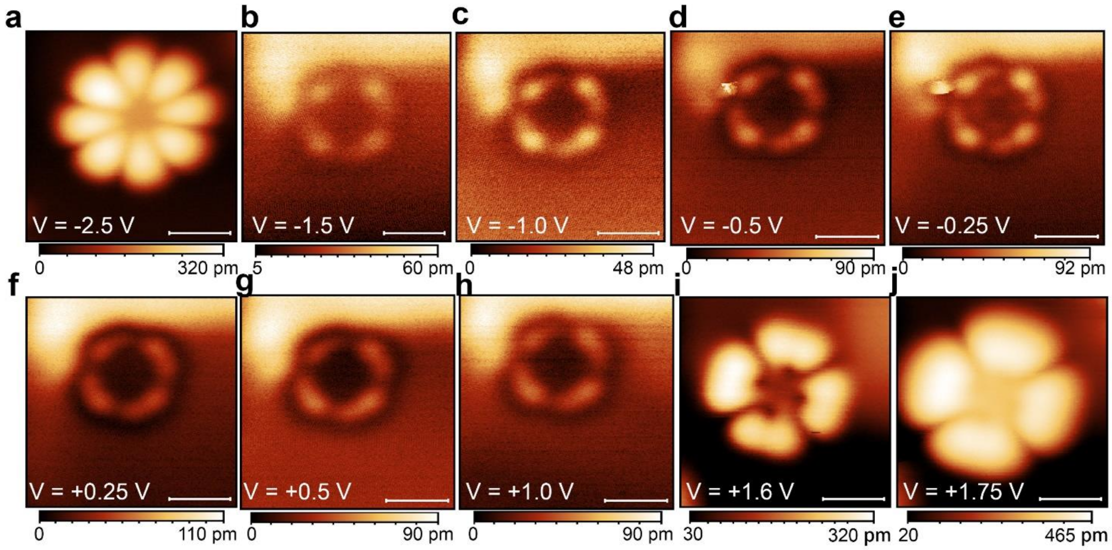

Figure S2. Constant current STM images acquired at negative (a-e) and positive (f-j) bias voltages of a single PtOEP molecule on 2ML NaCl/ Ag (111) (I = 2 pA). The corresponding tunneling voltage is indicated in each panel. The PtOEP molecule is stabilized by a step edge of NaCl, a fact that becomes apparent within the gap (panels b-h) due to the low profile of the in-gap structure compared to the HOMO. The height scales vary for the different panels of the figure as indicated below each image. Scale bars: 1 nm.

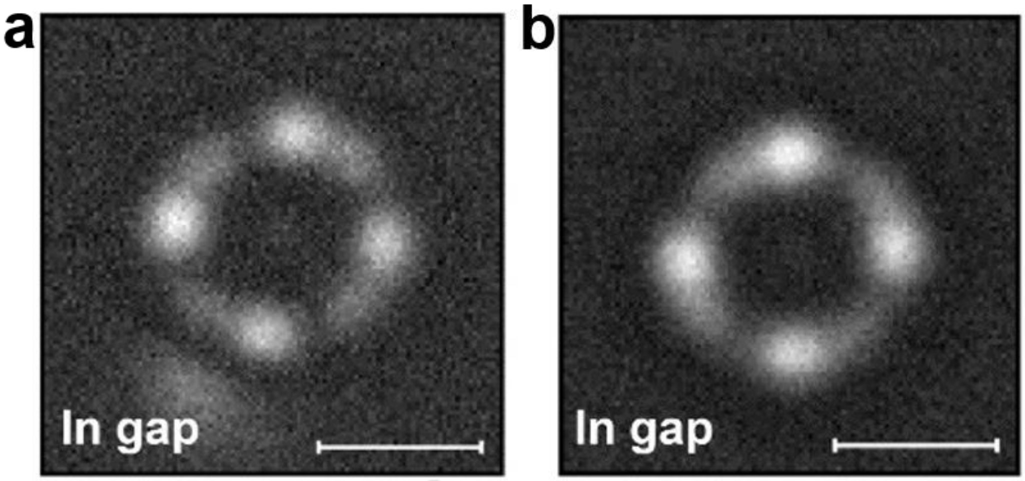

Figure S3. Constant-height STM images of PtOEP on 2ML NaCl on Au (111) (V = +0.3 V) demonstrating the existence of opposite chiralities. Note, that a and b are measured on different molecules and a change of chirality by STM manipulation was observed very rarely after employing high positive (> +2V) voltages. Scale bars: 1 nm.

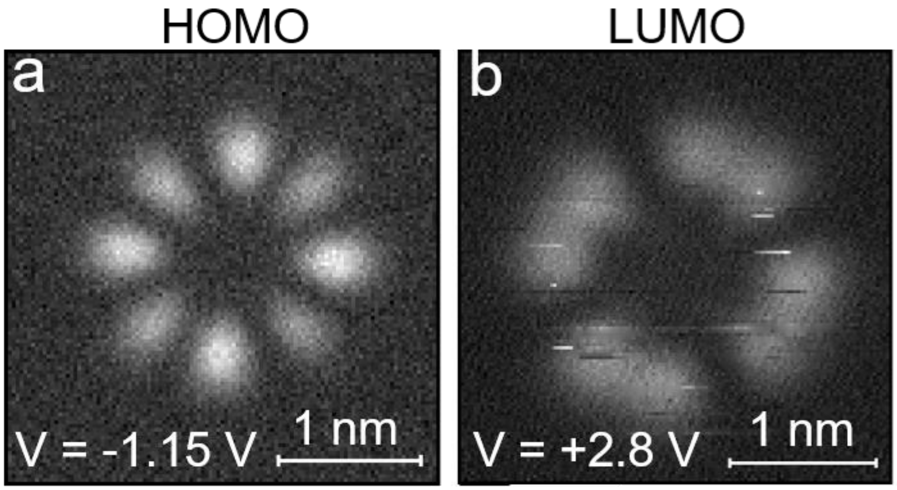

Figure S4. Constant-height STM images acquired at negative (a) and positive (b) bias voltages of PtOEP on 2ML NaCl on Au (111) corresponding to the HOMO and LUMO images, respectively. The corresponding tunneling voltage is indicated in each panel.

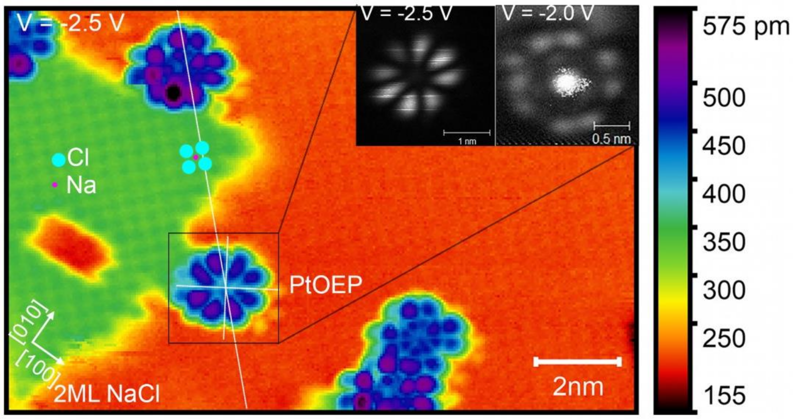

Figure S5. High-resolution STM image of PtOEP and ZnPc on 2 MLs of NaCl on Ag (111) acquired at V = -2.5 V, I = 2 pA. The underlying NaCl lattice is made visible due to the choice of color scale. The insets are zoomed-in constant-height STM images of the molecule inside the marked square frame, acquired at the indicated voltages. An evaluation similar to the one in Fig. 3 shows that this molecule is adsorbed with its metal core located on top of an Na ion.

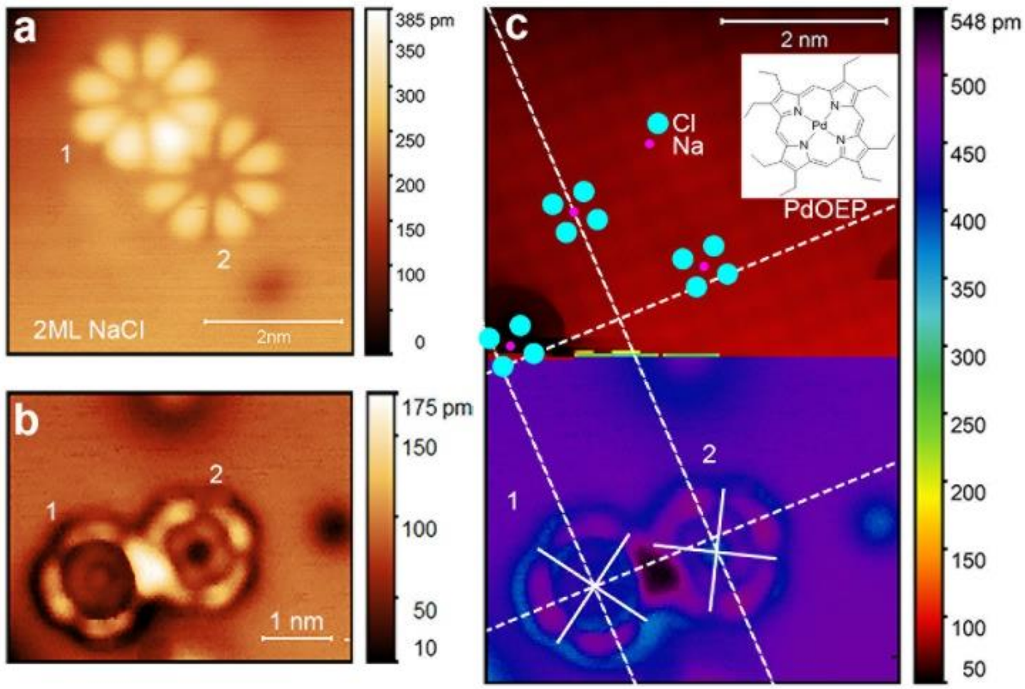

Figure S6. a. STM image of the HOMO of two PdOEP molecules adsorbed on 2 ML NaCl / Ag (100) (V = -2.5 V, I = 2 pA). b. 50° rotated STM image of the same two PdOEP molecules acquired in the transport gap, V = -1.5 V, I = 2 pA. c. In-gap STM image of the PdOEP molecules with the same image orientation as in (b). Scanning was interrupted in the middle of the image for a few seconds only to change tunnel parameters thus allowing to image the NaCl lattice and the in-gap structure in the same measurement (top part: V = -0.2 V, I = 30 pA; bottom part: V = -1.5 V, I = 1.2 pA). The white lines indicate the center and orientation of the molecules. The white dashed lines indicate the the atomic rows of the NaCl lattice. Note that in contrast to Fig. 3 in the main text, here the molecules are adsorbed on the NaCl layer that is imaged with atomic resolution.

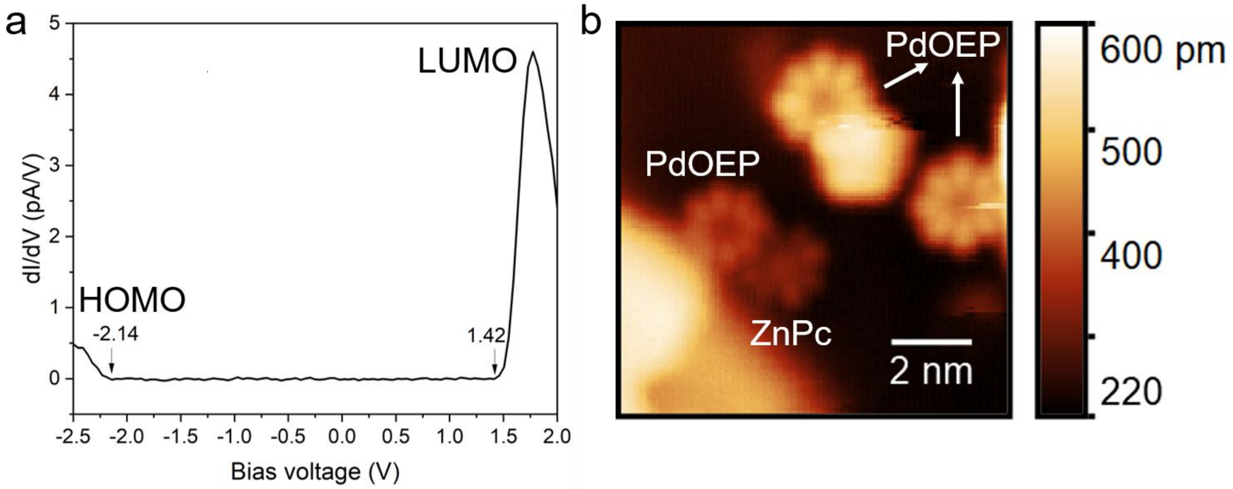

Figure S7 a. dI/ dV spectra of PdOEP on 2ML NaCl/ Ag (111) b. STM image of PdOEP and ZnPc molecules coadsorbed on 2ML NaCl/ Ag (111) (V = -2.5 V, I = 2 pA)

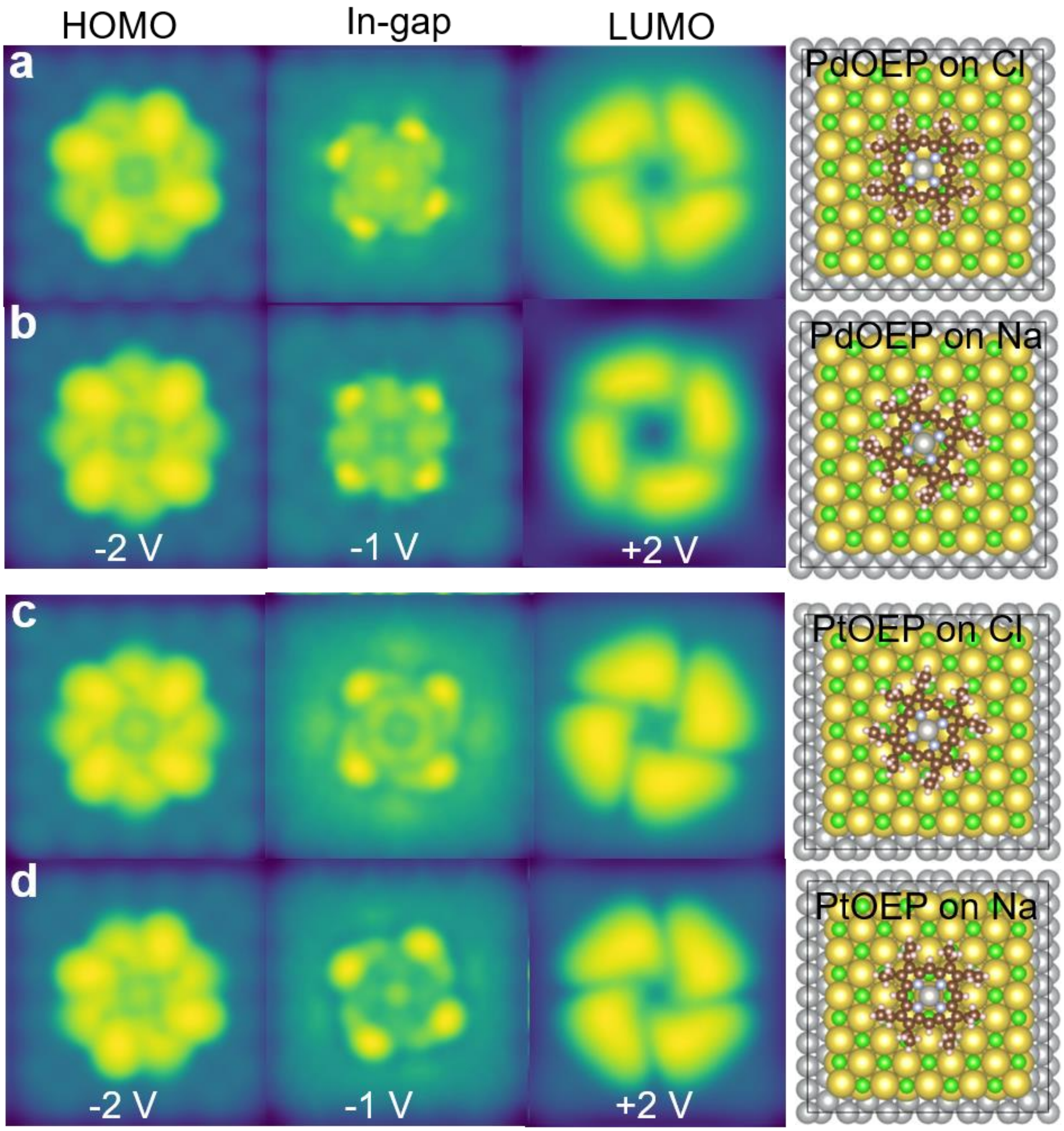

Figure S8. a,b. Simulated STM images of the frontier orbitals and in-gap state of PdOEP adsorbed on a Cl (a) and a Na ion (b) on 2 ML NaCl / Ag (100) and the corresponding atomic models. c,d. Simulated STM images of the frontier orbitals and in-gap state of PtOEP adsorbed on a Cl (c) and a Na ion (d) on 2 ML NaCl / Ag (111) and the corresponding atomic models.

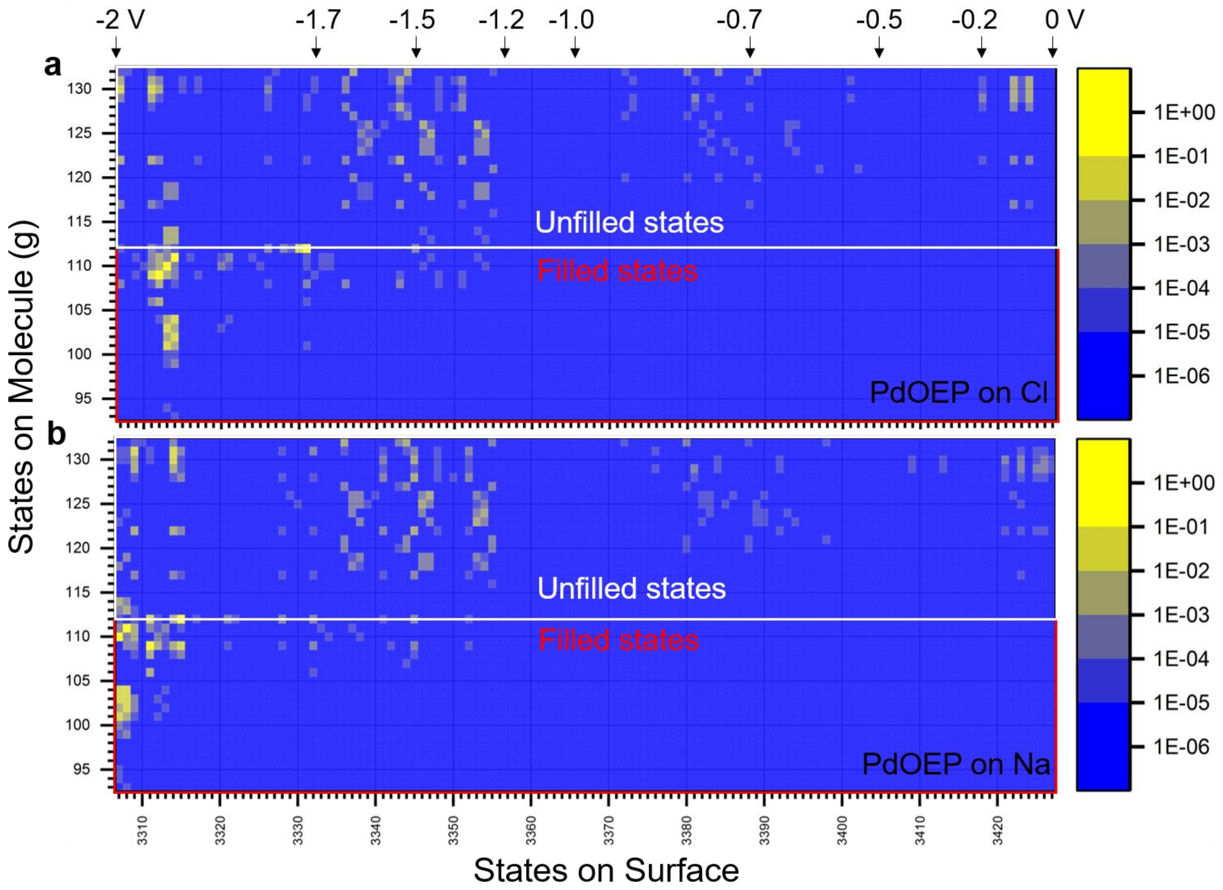

Figure S9. Overlap analysis of PdOEP on a Cl ion (a) and on a Na ion (b) on 2ML NaCl/Ag (100), showing that the character of in-gap states resembles unfilled orbitals of the molecule. The electronic states computed for PdOEP on surface are given in the x-axis, where the in-gap states are the states between 0 and -1.7 V (labeled as state 3427 to state 3332). These electronic states are compared with the electronic states of PdOEP in gas phase given in y-axis where HOMO, HOMO-1, … are labelled as state 112, 111, …, and LUMO, LUMO+1, … are labeled as 113, 114, … respectively. The z-axis (given as color bar) shows the degree of similarity between the electronic states of PdOEP on surface and in gas phase (computed by evaluating overlap integral between the two states, see section 1.2 above).

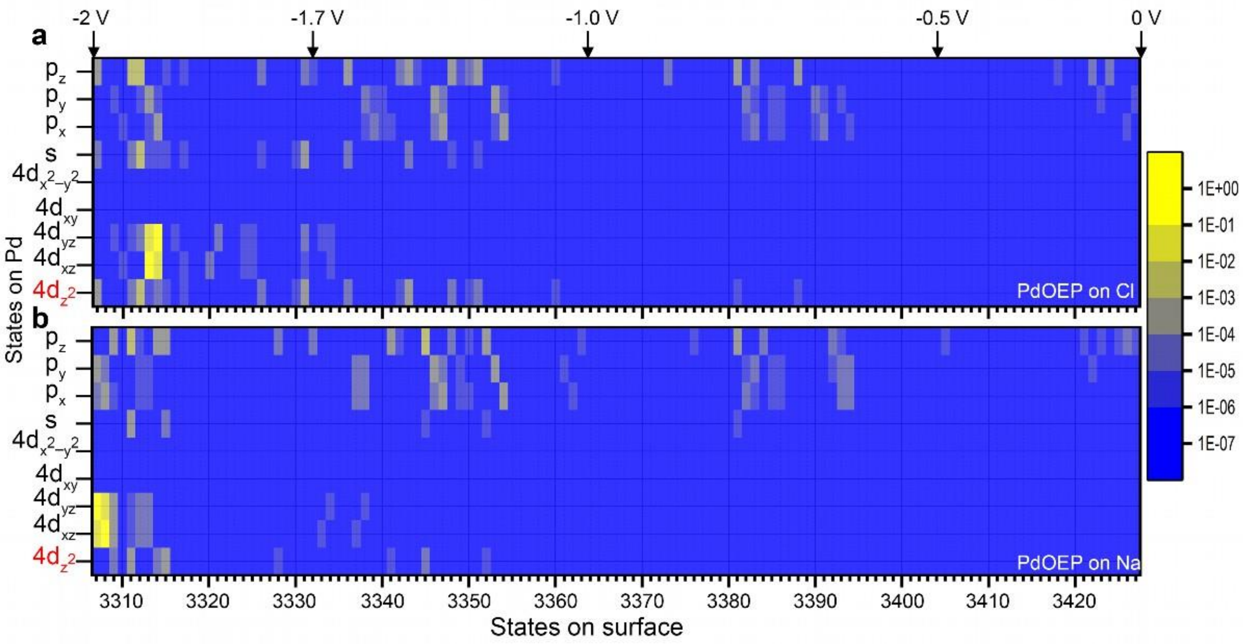

Figure S10. Overlap analysis of the states on the Pd atom of PdOEP and the surface states for PdOEP on a Cl ion (a) and on a Na ion (b) on 2ML NaCl/Ag (100), showing the large contribution of $4dz^2$ orbital to the in-gap states. The electronic states of PdOEP on surface are compared against various Pd 4d orbitals as well as Pd 5s and 5p orbitals (see section 1.2 above).

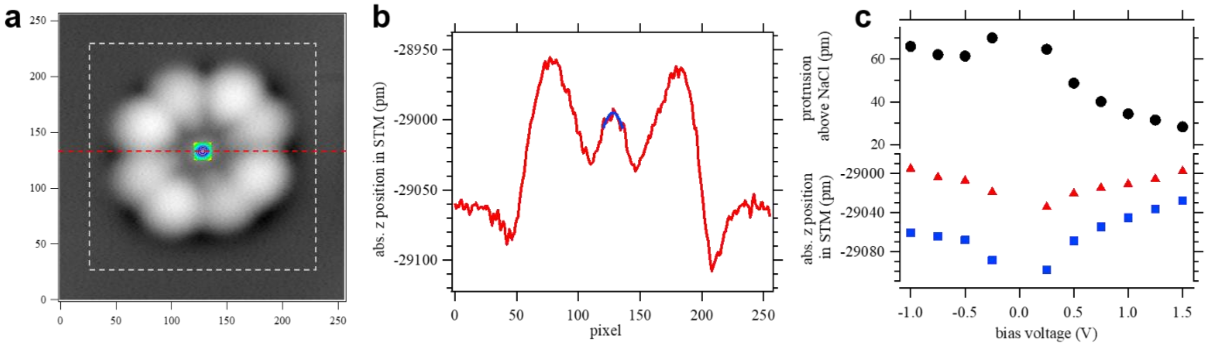

Figure S11. Evaluation of the central protrusion. (a,b) example for the data at -1 V bias voltage: (a) constant current topography (identical to Fig. 2, second image in bottom panel) and 2D Gaussian fit (central square) of the central protrusion (in rainbow colors: lines of constant height). (b) Cross section through the center of the molecule along the dashed red line in (a) and the corresponding Gaussian fit (blue curve). (c) z coordinate of the NaCl surface (blue squares) and of the central protrusion (red triangles). The difference between the red and blue data defines the *relative* height of the protrusion (black circles) with respect to the NaCl plane.

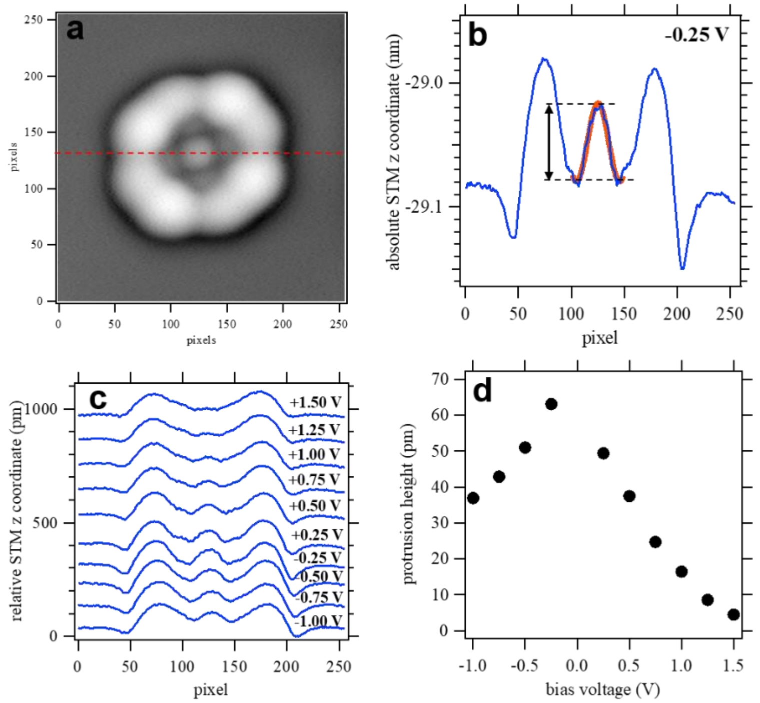

Figure S12. Alternative evaluation of the data in Fig. 2, where the protrusion height is determined with respect to the height of the dark ring around the molecule's center as illustrated in panel b. (a,b) Example for the data at -0.25 V bias voltage: (a) Constant current topography (identical to Fig. 2, second image in bottom panel). (b) Cross section along the dashed red line in panel (a)

and the corresponding fit (red curve). (c) Cross section through the center of the molecule at varied bias voltage. (d) Measured protrusion height as a function of bias voltage. We find a qualitatively similar behavior to that in Fig. S11, namely a negative slope as a function of bias voltage, overlaid with an increase of protrusion height around 0 V. Note that in Fig. 2 the evaluation of Fig. S11 is used because the NaCl plane provides a more defined reference than the dark ring in the molecule's center.

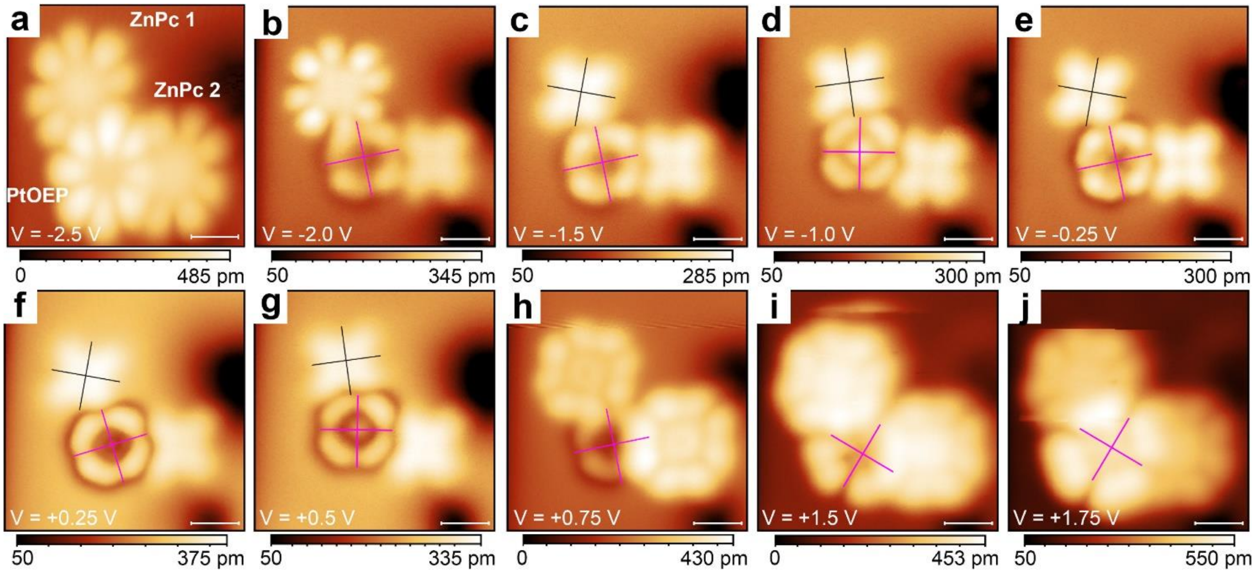

Figure S13: a-j. STM images acquired at negative (a-e) and positive (f-j) bias voltages of PtOEP and ZnPc molecules coadsorbed on 3ML NaCl / Ag (111) (I = 2 pA). The tunneling voltages are indicated in the lower left of each panel. Note, that chronologically, panels g and i were measured between panels d and e in the data sequence. Crosses mark the orientation of ZnPc (black) and PtOEP (red) molecules. Scale bars: 1 nm.

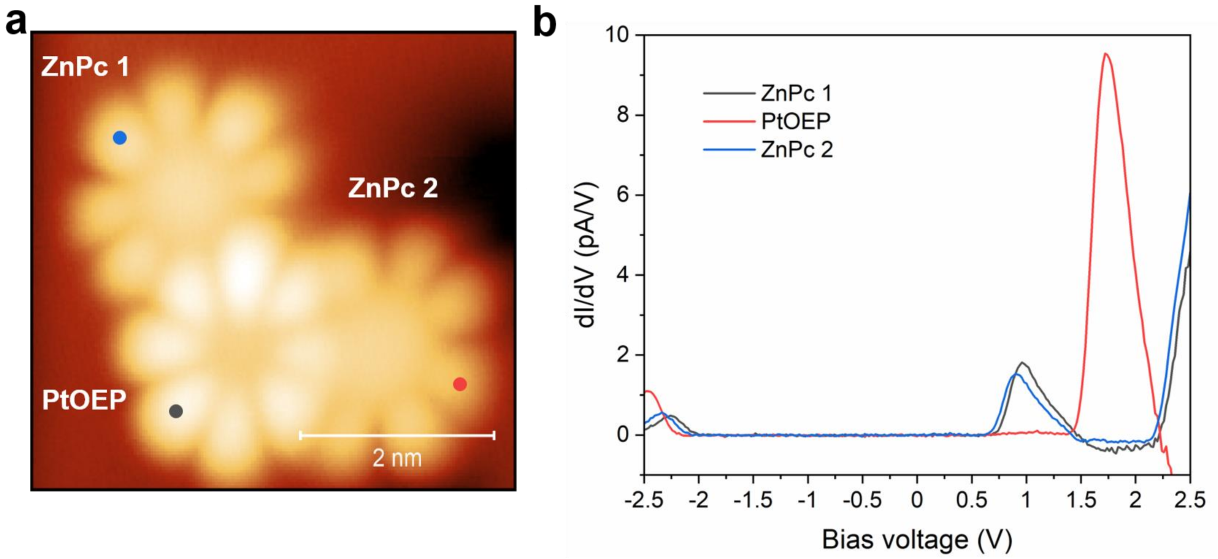

Figure S14. a. STM image of PtOEP and ZnPc molecules on 3 ML NaCl on Ag (111), V= -2.5 V, I= 2 pA. b. dI/ dV spectra of the PtOEP and the two labeled ZnPc molecules.

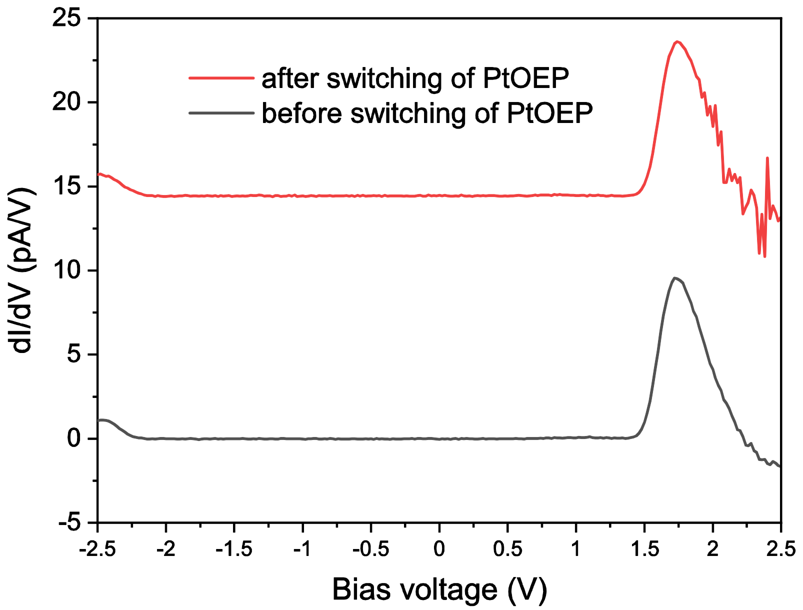

Figure S15. dI/ dV spectra of the PtOEP molecule shown in Figure 6 and Figure S12 recorded before (black) and after (red) switching adsorption site. The sharp spikes between 2 V and 2.5 V are a clear indication of the instability of the molecule. The STM image that was recorded after this bias spectrum measurement confirmed this finding (Fig. 6b and Figure S13d). The detail about the STM manipulation of PtOEP is shown in section 1.4 above.

3. References

1. Kuhnke, K.; Kabakchiev, A.; Stiepany, W.; Zinser, F.; Vogelgesang, R.; Kern, K. Versatile optical access to the tunnel gap in a low-temperature scanning tunneling microscope. *Rev. Sci. Instrum.* **2010**, *81* (11), 113102.

2. Stadler, J.; Schmid, T.; Zenobi, R. Nanoscale chemical imaging using top-illumination tip-enhanced Raman spectroscopy. *Nano lett.* **2010**, *10* (11), 4514-4520.

3. Yang, B.; Kazuma, E.; Yokota, Y.; Kim, Y. Fabrication of sharp gold tips by three-electrode electrochemical etching with high controllability and reproducibility. *J. Phys. Chem. C* **2018**, *122* (29), 16950-16955.

4. Momma, K.; Izumi, F. VESTA3 for three-dimensional visualization of crystal, volumetric and morphology data. *J. of Appl. Crystallogr.* **2011**, *44*, 1272–1276.

5. Kresse, G.; Hafner, J. Ab initio molecular dynamics for liquid metals. *Phys. Rev. B* **1993**, *47*, 558–561.

6. Kresse, G.; Furthmüller, J. Efficient iterative schemes for ab initio total-energy calculations using a plane-wave basis set. *Phys. Rev. B* **1996**, *54*, 11169–11186.

7. Blöchl, P. E. Projector augmented-wave method. *Phys. Rev. B* **1994**, *50*, 17953–17979.

8. Kresse, G.; Joubert, D. From ultrasoft pseudopotentials to the projector augmented-wave method. *Phys. Rev. B* **1999**, *59*, 1758–1775.

9. Grimme, S.; Antony, J.; Ehrlich, S.; Krieg, H. A consistent and accurate ab initio parametrization of density functional dispersion correction (DFT-D) for the 94 elements H-Pu. *The J. Chem. Phys.* **2010**, *132*, 154104.

10. Perdew, J. P.; Burke, K. & Ernzerhof, M. Generalized Gradient Approximation Made Simple. *Phys. Rev. Lett.* **1996**, *77*, 3865–3868.

11. Tersoff, J.; Hamann, D. R. Theory of the scanning tunneling microscope. *Phys. Rev. B* **1985**, *31*, 805–813.

12 Leon, C. C.; Grewal, A.; Kuhnke, K.; Kern, K.; Gunnarsson, O. Anionic character of the conduction band of sodium chloride. *Nat. Commun.* **2022**, *13*, 981.

13 Chen, H.-Y. T.; Pacchioni, G. Properties of Two-Dimensional Insulators: A DFT Study of Co Adsorption on NaCl and MgO Ultrathin Films. *Phys. Chem. Chem. Phys.* **2014**, *16*, 21838–21845.
